# Recording information on protein complexes in an information management system

**DOI:** 10.1016/j.jsb.2011.05.009

**Published:** 2011-08

**Authors:** Marc Savitsky, Jonathan M. Diprose, Chris Morris, Susanne L. Griffiths, Edward Daniel, Bill Lin, Susan Daenke, Benjamin Bishop, Christian Siebold, Keith S. Wilson, Richard Blake, David I. Stuart, Robert M. Esnouf

**Affiliations:** a Division of Structural Biology, Wellcome Trust Centre for Human Genetics, University of Oxford, Roosevelt Drive, Oxford OX3 7BN, UK; b The Oxford Protein Production Facility UK, Research Complex at Harwell, Rutherford Appleton Laboratory, R92, Harwell Oxford, Didcot OX11 0FA, UK; c CSED, STFC Daresbury Laboratory, Warrington WA4 4AD, UK; d York Structural Biology Laboratory, Department of Chemistry, University of York, Heslington, York YO10 5DD, UK; e Diamond Light Source Ltd., Diamond House, Harwell Science and Innovation Campus, Didcot OX11 0DE, UK; f Wellcome Trust Centre for Human Genetics, University of Oxford, Roosevelt Drive, Oxford OX3 7BN, UK

**Keywords:** Laboratory Information Management System (LIMS), Informatics, Data management, Data model, Protein complex

## Abstract

The Protein Information Management System (PiMS) is a laboratory information management system (LIMS) designed for use with the production of proteins in a research environment. The software is distributed under the CCP4 licence, and so is available free of charge to academic laboratories. Like most LIMS, the underlying PiMS data model originally had no support for protein–protein complexes. To support the SPINE2-Complexes project the developers have extended PiMS to meet these requirements. The modifications to PiMS, described here, include data model changes, additional protocols, some user interface changes and functionality to detect when an experiment may have formed a complex. Example data are shown for the production of a crystal of a protein complex. Integration with SPINE2-Complexes Target Tracker application is also described.

## 1 Introduction

Recent years have seen the development of many laboratory information management systems (LIMS), The majority of such LIMS are developed commercially and/or devoted to relatively simple process management, for examples see http://limsource.com/products/vproduct.html (2010). Academically developed LIMS related to structural biology include LISA (Haebel et al., 2001), XTRACK (Harris and Jones, 2002), SESAME (Zolnai et al., 2003), CLIMS (Fulton et al., 2004), HALX (Prilusky et al., 2005) and MOLE (Morris et al., 2005). The Protein Information Management System (PiMS) is an academically funded LIMS focused on the management of data describing the production and crystallization of proteins in a research environment (Morris et al., 2011). PiMS was originally developed as part of the UK BBSRC’s SPoRT initiative and is now principally supported by the CCP4 project (Collaborative Computational Project, Number 4, 1994). PiMS is distributed under the CCP4 licence and, therefore, is free to any laboratory (commercial or academic) with a CCP4 licence. Details of licensing and downloading PiMS can be found on the project web site, http://www.pims-lims.org/.

PiMS is a fully featured and flexible LIMS, but it is built around a small number of central concepts that are already familiar to practitioners in the field: *Target*, *Construct*, *Sample*, *Experiment* and *Protocol*.

A *Target* represents a single biological macromolecule (usually a protein) whose study is the purpose of some research. In structural genomics, the biological entities of interest have been the open reading frames (ORFs) of organisms and the proteins that each of them encodes. This two-sequence model of a *Target* has been incorporated into PiMS.
A *Construct* in PiMS is a record of what the scientist intends to express, including sequence mutations, truncations, fusions and affinity tags. Scientists usually design multiple constructs in their attempts to express each *Target*. PiMS facilitates the design and recording of *Constructs* by recommending PCR primer sequences and maintaining lists of standard primer extensions.
A *Sample* is intended to represent any physical sample. It may contain molecules related to a *Target* or *Construct* (in the form of DNA or protein), or it may represent a reagent that has been produced in (or brought into) the laboratory.
An *Experiment* describes how *Samples* were produced and acted on, and relates to a particular *Construct*. *Samples* are the optional inputs to and outputs from *Experiments*, thus allowing complicated workflows to be built up.
A *Protocol* is a reusable template from which *Experiments* can be created. A set of *Experiments* based on a single *Protocol* can be grouped together, most usefully to represent plate-based experiments.

Many proteins are biologically active only in a complex with other proteins (*e.g.* Gavin et al., 2006) and so many research projects, including the SPINE2-Complexes project, have research objectives which are not adequately represented by a single ORF product. To record work on complexes some modifications to PiMS are required. However, the representation of complexes presents some design challenges:

• Is a complex something that is hypothesised to exist naturally in biology or any set of expressed constructs that interact?
• Should one distinguish between, for example, two proteins in a complex and the same two proteins merely co-existing in a solution?
• Is it valuable to distinguish between monomers and homodimers for a single protein species?
• How should LIMS deal with the programmatic and user-interface complexity introduced by the many-to-many relationships required to describe complexes?
• How are complexes “related” to each other for searching and comparison purposes?

In dealing with these issues the PiMS developers were guided by the SPINE2-Complexes Target Tracker (see below) which, in turn, drew on and extended the model developed for the 3D-Repertoire project (Romier et al., 2006). This technical note describes the data model design decisions that were taken as well as outlining the other changes within PiMS that were required to manage this extra richness: the definition of new *Protocols* specific to creating complexes, how the user interface changes were kept to a minimum so as not to impact on non-complex work and the development of new functionality to recognise when experiments were intended to form potential complexes. Finally, these changes are illustrated by considering the use of PiMS to record the creation of a real protein complex crystal.

## 2 Methods

**Fig.1:**
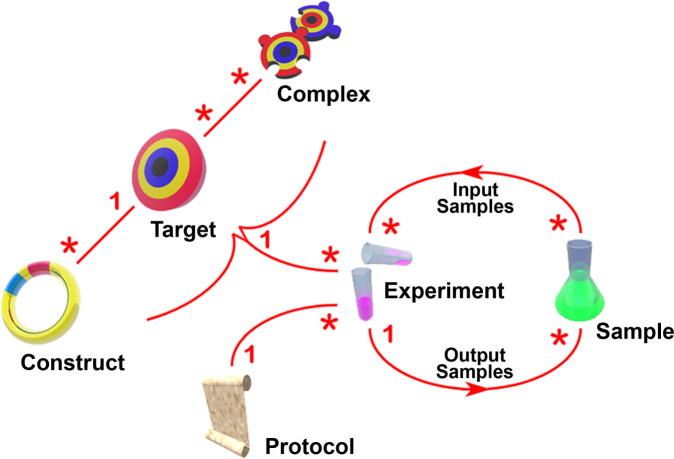
A schematic showing the relationships between the core PiMS concepts relevant to the recording of work on complexes. The “1”s and “*”s on the red lines indicate one-to-many and many-to-many relationships, respectively. For example, a *Construct* belongs to a single *Target* but a *Target* may be a component of many *Complexes*. The icons are used throughout the PiMS user interface to indicate the relevant object types. An additional many-to-many relationship exists between *Sample* and *Construct* but is not relevant to the work described here.

The PiMS data model was changed to allow a collection of macromolecules to be recorded as the target of research. This new concept is called the PiMS *Complex* and it is intended to represent a biologically relevant complex, *i.e.* between two or more naturally occurring proteins. Thus, a group of *Targets* describing individual proteins (and any small molecule ligands) defines a *Complex*. If a complex is studied by making multiple constructs for each component protein then all complexes between expressed constructs are linked to the same *Complex*. This reduces the number of complexes that have to be described and helps in detecting relationships between complexes (*i.e.* common components in multiple complexes). This implementation minimises the impact on existing PiMS use: the individual proteins of a complex are often research targets in their own right and producing a protein complex often involves producing the individual proteins prior to combination. A second data model change was also required: to allow an *Experiment* to relate to a *Complex* rather than to a *Construct* when appropriate. This second change was more difficult to implement as it required significant changes to the internal organisation of PiMS, however these changes were hidden from the user. The central PiMS concepts and their revised relationships are shown schematically in Fig. 1.

New user-interface components that allow users to create and search *Complexes* have been added under the “Target” menu in PiMS. There are only two required pieces of information for a new *Complex*, its *Name* and a description. *Targets* can be added to, and removed from, a *Complex* through a simple search-and-select interface, with recently viewed *Targets* shown at the top of the list for convenience.

Extra links have been provided on the PiMS *Experiment* and *Sample* pages to allow users to navigate easily back to the parent *Complex(es)*. All laboratory activities are recorded as *Experiments*. Each *Experiment* has some general information (its status, who ran it, when it was run, whether it worked, which *Construct*, *Target* and now *Complex* it relates to, etc.) and some specific information (what *Samples* went in, what *Samples* were produced and runtime parameters/results such as PCR annealing temperature). A *Sample* produced by one *Experiment* goes on to be used as an input to another *Experiment* and the production history of any *Sample* can be obtained by retracing this chain. Since a PiMS *Sample* can represent any experimental product – including a single protein or a protein complex – recording an *Experiment* involving a complex is no different to recording any other *Experiment*.

A PiMS *Protocol*, a reusable *Experiment* template, records the categories of *Samples* that are allowed as inputs and produced as outputs amongst other things. Importantly, PiMS avoids the use of special categories for dealing with protein complexes: a PiMS *Plasmid* describes a plasmid whether it is bi-cistronic or not; a PiMS *Soluble Protein* could equally well refer to a single protein (purified or not), multiple proteins, a well-defined protein–protein complex or some combination of these species. In this way PiMS avoids a difficult (and potentially artificial) distinction: whether mixing two protein components gives a mixture or a complex. Indeed, even with a single protein species there is ambiguity since its oligomeric state may vary over time or as a function of concentration.

**Fig.2:**
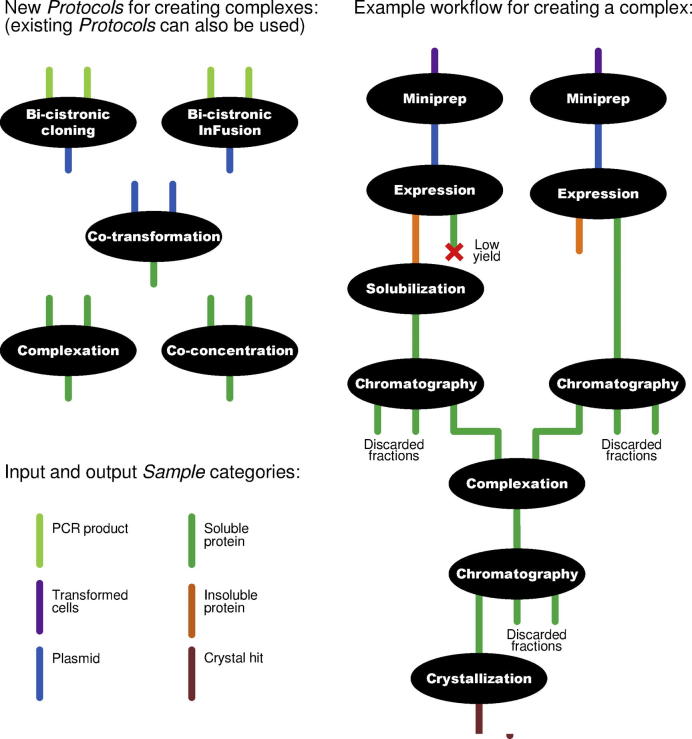
A schematic showing the new standard PiMS *Protocols* for working with complexes (left-hand side) and an example of how *Experiments* based on these *Protocols* fit together to build up a workflow (right-hand side). *Protocols* specify the categories of *Samples* that are allowed as inputs and produced as outputs (colour-coded in the diagram). *Experiments* “snap together” by matching one output category to the next input category. By specifying identical input categories, a *Protocol* allows unrelated *Samples* of the same type to be combined, shown by the “Complexation” step in the example workflow.

Existing *Protocols* are just as valid for complexes as for single proteins. However, as part of the work described here, the standard set of PiMS *Protocols* was augmented by five new *Protocols* which describe *Experiments* uniquely relevant to work on complexes: two for bi-cistronic expression of two products from a single vector; one for co-expression of two products by co-transformation of two plasmids into one cell; one for mixing of two separately expressed products (“Complexation”); and one for co-concentration of two separately expressed products. A characteristic of these *Protocols* is that they specify two or more input *Samples* with the same category. For example, the Complexation *Protocol* specifies two input *Samples* belonging to category *Soluble Protein* (Fig. 2).

The flexibility of the *Protocol* system combined with the dynamic generation of production history means that no further changes are required for PiMS to be used to record the production of complexes. PiMS has a user interface that allows *Protocols* to be created, copied, edited and deleted making it straightforward to create new *Protocols* describing novel methods of complex formation.

## 3 Results and discussion

Although significant in design terms, recording protein complexes is only a small part of PiMS usage. Therefore, it was essential that introducing this functionality should not make PiMS any harder to use. This goal was achieved primarily due to the flexible nature of the PiMS *Protocol* system and its approach to *Sample* typing. Furthermore, these features make PiMS just as easy to use for recording and reporting work on complexes as single proteins, as illustrated by the following examples.

### 3.1 Production of a Hedgehog/Hedgehog Interacting Protein Complex

Hedgehog (Hh) family proteins are ubiquitous in tissue growth, patterning and morphogenesis. Dysregulation of Hh signalling can have severe pathological consequences and is an intensely active field of research. Several cell surface receptors, for example Hedgehog Interacting Protein (HIP), transduce and/or regulate Hh signals. The Hh–HIP interaction has been implicated in neuronal pathway development as well as stem cell maintenance and cancer. Bishop et al. (2009) undertook a structural study of the Hh–HIP complex as part of the SPINE2-Complexes project and their work was recorded in PiMS using the new functionality for complexes. The Hh–HIP complex was produced by separate expression and purification of the components followed by mixing to form the complex and a further purification step prior to crystallisation trials.

The project was used as a test case for the new features of PiMS and the new *Protocols* were created as part of this process. The “Complexation” *Protocol* was developed for the key complex-formation step and has two input *Samples* and one output *Sample*, all of type *Soluble Protein* (Fig. 2). A total of 25 different *Experiments* were recorded in PiMS to account for the whole process from construct design to crystal growth. After minimal training, a research student was able to record the work (and even contribute to defining the new *Protocols*) within PiMS. For navigating across, and keeping track of, the complicated multi-threaded workflow that was generated, the interactive diagram features of PiMS (Morris et al., 2011) were found to be particularly useful.

**Fig.3:**
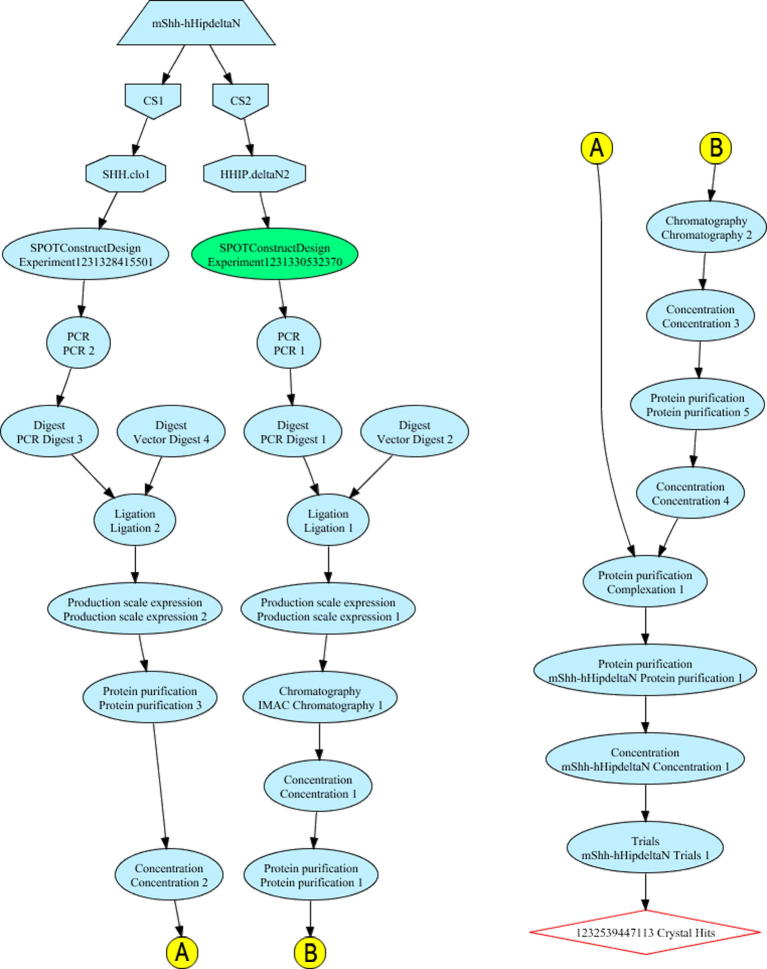
Screenshot of the Sample History Report diagram for the production history of Hh–HIP complex crystal (Bishop et al., 2009). The trapezoid at the top represents the *Complex*; pentagons represent *Targets*; octagons represent *Constructs*; ellipsoids represent *Experiments* and the red diamond at the end represents the *Sample* whose production history is shown. All other *Samples* are represented by the lines between *Experiments*. Within PiMS the diagram is interactive: clicking on one of the shapes will take you to the relevant page in PiMS. The diagram has been reformatted for publication by splitting in half and introducing the yellow A and B continuation markers.

The whole project history can be summarised clicking on the PiMS “Sample History Report” button, which appears near the top of every *Sample* page (Morris et al., 2011), for the resulting crystal hits. This one-page report details the full production history that led to that *Sample*. It can include all the *Complexes*, *Targets*, *Constructs*, *Samples*, *Experiments* and *Results* involved in the workflow and present these both in tabular form and as a workflow diagram. For the Hh–HIP example, the summary workflow diagram (*i.e.* just the *Complexes*, *Targets*, *Constructs* and *Experiments*) is shown in Fig. 3. Such reports assist in the write-up of the work, giving a clear view of the successful workflow from amongst many possible dead ends and failures. Indeed, the long-term goal is for such a report to generate the “Materials and methods” for publication directly, although at the present time the exported PDF version of the report is probably more suited to inclusion as Supplementary information. The URL of the report can be directly shared with others, subject to PiMS’ access control, thus providing an efficient progress report for colleagues, collaborators and principal investigators.

### 3.2 Recording and representing data for many complexes: SPINE2-Complexes Target Tracker

**Fig.4:**
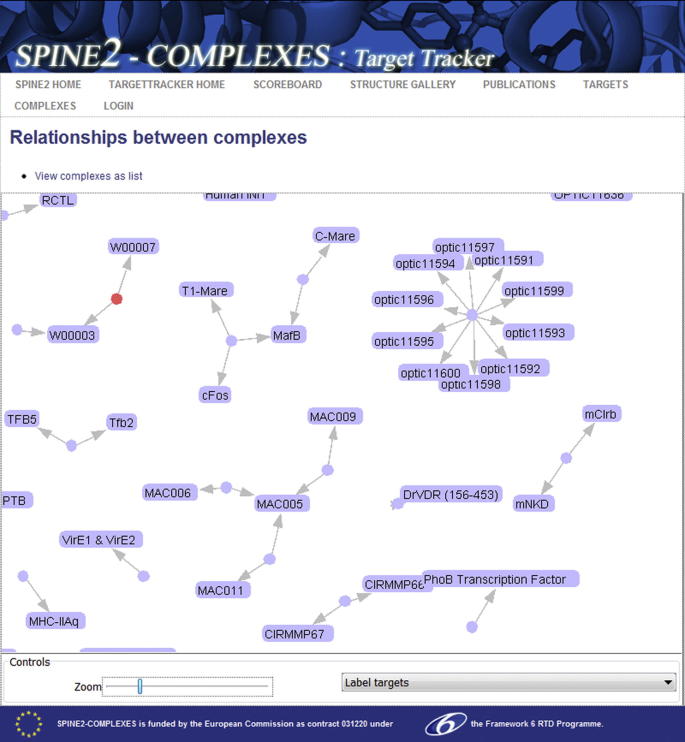
Screenshot from the SPINE2-Complexes Target Tracker graphical view of complexes (http://www.spine2.eu/SPINE2TT/ComplexMap.jsf, 2011). Circles represent complex targets and rectangles show the names of the component proteins. Note that proteins such as MAC005 and MafB are components of multiple, hence related, complexes. The view can be manually rearranged using drag-and-drop functionality and clicking on a complex or target reports the progress toward that component.

Structural genomics and proteomics initiatives have, for some time, coordinated and publicised their work by reporting to a central registry, TargetDB (Chen et al., 2004; http://targetdb.pdb.org/, 2010). TargetDB uses an ORF-based definition of a target which is not well suited to recording protein complexes and so the SPINE2-Complexes project developed its own registry, called “Target Tracker” (http://www.spine2.eu/SPINE2TT/, 2010). The Target Tracker enables project members to enter the list of complexes on which they were working, to report progress toward production and characterisation of those complexes (and their components) and to compare related complexes across the project. While the majority of data were entered manually, Target Tracker includes an interface that allows for bulk upload of data from an XML document and a specification of the document’s schema. One goal of Target Tracker development was to provide a graphical interface for showing the project’s status (Fig. 4; http://www.spine2.eu/SPINE2TT/ComplexMap.jsf, 2011). This view is created on the fly by a dynamic Java applet and can be scaled and manipulated by user. Complexes and their components are tied together by arrows and the layout is controlled by a pseudo-force field derived from sequence similarity.

Support for Target Tracker was added to PiMS as part of the SPINE2-Complexes project. The Sample History Report already allowed the export of a PDF report, and this functionality was extended to enable the export of a Target Tracker-compliant XML document. An additional button (labelled “Export to Spine2”) is present on the Sample History Reports for those *Samples* which are linked to *Complexes*. While PiMS is able to infer the potential existence of an unrecorded *Complex* from a *Sample’s* production history, the decision was taken to require manual declaration of *Complexes* prior to exporting data to Target Tracker. Therefore, additional checking is required in PiMS to ensure that all production paths share a common predefined *Complex*.

While the exported XML is in Target Tracker’s format, the schema was largely derived as an extension to and expansion of that of TargetDB at the time (version 1, http://targetdb.sbkb.org/target.dtd, 2011). The XML could be post-processed to match the current TargetDB schema (version 2, http://targetdb.sbkb.org/TargetDB/documentation/targetdb.v2.dtd, 2011) for uploading both the complex and its individual components to TargetDB.

## 4 Conclusion

Few LIMS have specific features for recording protein complexes and dealing with the extra data model richness they introduce. To the best of our knowledge, the extensions of the PiMS LIMS described here make it uniquely suited to this task and the way these extensions have been implemented has not introduced undue complexity for users. In PiMS, it is as easy to work with complexes as it is to work with single protein species. We have demonstrated the utility of this new functionality in recording the production of a protein complex from the field of cell signalling. Furthermore, by showing how PiMS can export data to the SPINE2-Complexes Target Tracker we have provided a model for data exchange with other applications. PiMS is available from the project website (http://www.pims-lims.org/, 2010) and is distributed under the CCP4 licence (http://www.ccp4.ac.uk/ccp4license.php, 2010).
